# Novel ultrasound neuromodulation therapy with transcranial pulse stimulation (TPS) in Parkinson’s disease: a first retrospective analysis

**DOI:** 10.1007/s00415-023-12114-1

**Published:** 2023-11-30

**Authors:** Sarah Osou, Sonja Radjenovic, Lena Bender, Martin Gaal, Anna Zettl, Gregor Dörl, Eva Matt, Roland Beisteiner

**Affiliations:** 1 Department of Neurology, Medical University of Vienna, Spitalgasse 23, 1090 Vienna, Austria; 2 Department of Pediatrics and Adolescent Medicine, Medical University of Vienna, Währinger Gürtel 18-20, 1090 Vienna, Austria

**Keywords:** Ultrasound, Neuromodulation, Transcranial pulse stimulation, Parkinson’s disease, Brain stimulation, Placebo

## Abstract

**Background:**

Transcranial Pulse Stimulation (TPS) has been recently introduced as a novel ultrasound neuromodulation therapy with the potential to stimulate the human brain in a focal and targeted manner. Here, we present a first retrospective analysis of TPS as an add-on therapy for Parkinson’s disease (PD), focusing on feasibility, safety, and clinical effects. We also discuss the placebo response in non-invasive brain stimulation studies as an important context.

**Methods:**

This retrospective clinical data analysis included 20 PD patients who received ten sessions of TPS intervention focused on the individual motor network. Safety evaluations were conducted throughout the intervention period. We analyzed changes in motor symptoms before and after TPS treatment using Unified Parkinson’s Disease Rating Scale part III (UPDRS-III).

**Results:**

We found significant improvement in UPDRS-III scores after treatment compared to baseline (pre-TPS: 16.70 ± 8.85, post-TPS: 12.95 ± 8.55; *p* < 0.001; Cohen’s d = 1.38). Adverse events monitoring revealed no major side effects.

**Conclusion:**

These preliminary findings suggest that TPS can further improve motor symptoms in PD patients already on optimized standard therapy. Findings have to be evaluated in context with the current literature on placebo effects.

**Supplementary Information:**

The online version contains supplementary material available at 10.1007/s00415-023-12114-1.

## Introduction

Non-invasive brain stimulation (NIBS) holds potential for alleviating motor symptoms of Parkinson’s disease (PD) [1]. Recently, a novel NIBS-technique called Transcranial Pulse Stimulation (TPS) has been introduced, which uses ultrasound pressure pulses to modulate brain activity [2]. TPS bears the advantage that the small ultrasound foci are independent from pathological conductivity changes and specific brain areas can therefore be precisely targeted, even in the depth of the brain [3, 4]. Although the field is still young, navigated ultrasound stimulation has shown promise in other neurological conditions [5], such as Alzheimer’s disease (AD) [5, 6], disorders of consciousness [7, 8], and depression [6, 9], and might be a valuable adjunct treatment for Parkinson’s disease.

The underlying mechanisms of ultrasound neuromodulation are not yet fully elucidated. According to previously published investigations, ultrasound pulses impact mechanosensitive ion channels. Thereby, the mechanical stimuli are transduced into biochemical signals, subsequently triggering downstream signal responses, and resulting in protein-level changes. [10, 11]. Recently, the mechanosensitive ion channel Piezo1 was identified as a significant mediator of the neuromodulatory impact of ultrasound in vivo [12]. Additionally, recent data indicate that single ultrasound pulses generate supra-threshold neuronal excitation [13]. However, further research is needed to understand how these biological effects translate into clinical outcomes and to determine the therapeutic effects of TPS.

In this paper, we present the first major retrospective clinical data analysis on ultrasound neuromodulation as an add-on therapy in patients with PD. The purpose of the study was to investigate the feasibility, safety, and clinical effects of TPS on motor symptoms. Considering that placebo effects have been shown to be particularly important in PD [14, 15], the results will be discussed in context of a comprehensive literature analysis on possible NIBS sham effects.

## Methods

### Study design

This was an open-label, uncontrolled, retrospective study to investigate the following questions: (i) is TPS safe and feasible in a broad uncontrolled spectrum of PD patients as typically seen in clinical practice, and (ii) are there indications for clinical effects as examined by clinical scores? The primary outcome measure was the change in the Unified Parkinson’s Disease Rating Scale part III (UPDRS-III) after completion of TPS treatment compared with pre-treatment scores.

### Patient sample

20 consecutive patients with a primary diagnosis of PD (various subtypes and co-morbidities) diagnosed by external specialists in neurology were included (15 men, 5 women; mean age 67.6 ± 7.5 years; age range 48–84 years; mean disease duration 53.5 ± 28.0 months; disease duration range 3–148 months). All patients had requested TPS treatment as add-on therapy (therapeutic attempt) and received ten sessions of TPS intervention within 2 weeks at the TPS Therapy and Development Center in Vienna (Austria). All patients were on state-of-the-art treatments optimized for the individual case by the treating neurologists. Patients were instructed not to change their optimized treatments during TPS therapy. Common inclusion criteria were written treatment request, clinical stability, the completion of the UPDRS assessment before and after therapy by external neurologists, and written informed consent. As required by practical clinical therapy, co-morbidities outside TPS contraindications were allowed. A detailed list of co-morbidities can be found in Table S2 in the Supplementary Information (SI). Common exclusion criteria were TPS contraindications (i.e., thrombosis, pregnancy, epiphyseal plates in children, tumor in the treatment area, cortisone treatments within 6 weeks before the first application, metal objects in the head, and pacemakers not approved for TPS^®^ therapy) as specified in the documentations of the NEUROLITH TPS system (Storz Medical AG, Tägerwilen, Switzerland).

### TPS parameters

Brain stimulation was performed using the NEUROLITH TPS system (Storz Medical AG, Tägerwilen, Switzerland) and the methodology developed by our research group over the past decade [2, 5]. The treatment protocol for PD encompassed ten TPS sessions conducted daily over a 2-week period, from Monday to Friday. In case of holidays occurring during the treatment weeks, two sessions were administered in a single day, each with reduced energy settings. Further details regarding the energy settings adjustments and impacted patients can be accessed in Table S1 in the SI. Each treatment session lasted approximately 30–45 min. All enrolled patients completed all ten treatment sessions. Immediately before treatment start, high-resolution magnetic resonance imaging (MRI) scans for exclusion of contraindications, judgement of brain morphology/brain pathology, and TPS navigation were recorded. In addition, each patient had a specific functional neurological investigation to evaluate the individual clinical state. A neurologist and clinical neuroscientist (R.B.) defined the individual target areas for TPS stimulation on these MR images. Motor network stimulation was focused on the primary sensorimotor area, supplementary motor area, and cingulate motor area. Depending on symptomatology (including cognitive deficits), additional target areas were included according to current state of the clinical neuroscientific literature (e.g., left dorsolateral prefrontal cortex for depression). By default, 4000 ultrashort (about 3 μs) ultrasound pressure pulses (energy flux density = 0.25 mJ/mm^2^ and pulse repetition rate = 4 Hz) were applied in each TPS session. Real-time tracking allowed for precise targeting and even distribution of pulses within the individualized target areas. In the context of clinical therapy, individualized treatment settings and parameter adjustments are essential. In the present study, one patient received a reduced energy level for subjective comfort, and nine patients received a 50% reduction in dose for the initial TPS session to allow for treatment adaptation. The individual treatment parameters are detailed in Table S1 in the SI.

### Patient safety evaluations

Adverse events (AE) were monitored during the 2 weeks of TPS therapy. At each visit, patients were asked to describe AE that occurred after the previous treatment session. Additionally, at the end of each TPS session, patients evaluated their pressure and pain experience during the treatment using visual analogue scales (VAS; 0 = none and 10 = very strong pressure/pain).

### Clinical evaluation and statistics

Patients underwent clinical evaluation within a 4-week window before and after the ten sessions of TPS intervention. On average, these clinical assessments occurred 14 and 13 days before the first and after the last TPS session, respectively. A detailed presentation of the individual time intervals between TPS therapy and clinical testing can be found in Table S1 in the SI. All clinical scores were assessed during the patients’ “ON” state by independent external neurologists. UPDRS-III was used to assess a change in the motor status, as the primary outcome measure. Importantly, two different versions of the UPDRS were used by independent neurologists, namely UPDRS and the revised MDS-UPDRS (revision of the UPDRS by the Movement Disorder Society) [16]. To enable a consistent analysis of UPDRS-III, the points of the supplementary items of the MDS-UPDRS-part III were removed. All statistical analyses were performed using IBM SPSS Statistics (version 28). Primary outcome scores were checked for normality and subsequently a two-sided, paired t test was performed. Effects were considered statistically significant if a *p* value < 0.05 was found.

## Results

### Patient safety evaluation and adverse events

Patient evaluations during the period of TPS intervention did not show any serious side effects. In total, 13 patients (65%) reported at least one mild AE during the 10 days of TPS treatment. Fatigue, headache, and dizziness were the most common AE and reported by 10 (50%), 6 (30%), and 6 (30%) patients, respectively. All events resolved within a day. VAS evaluation (0–10) of within-treatment pressure experience resulted in 91.5% VAS 0, 3.5% 1–3, 4% 4–6, and 1% 7–8 (percentages calculated over all TPS sessions).

### Motor scores: UPDRS-III

**Fig. 1:**
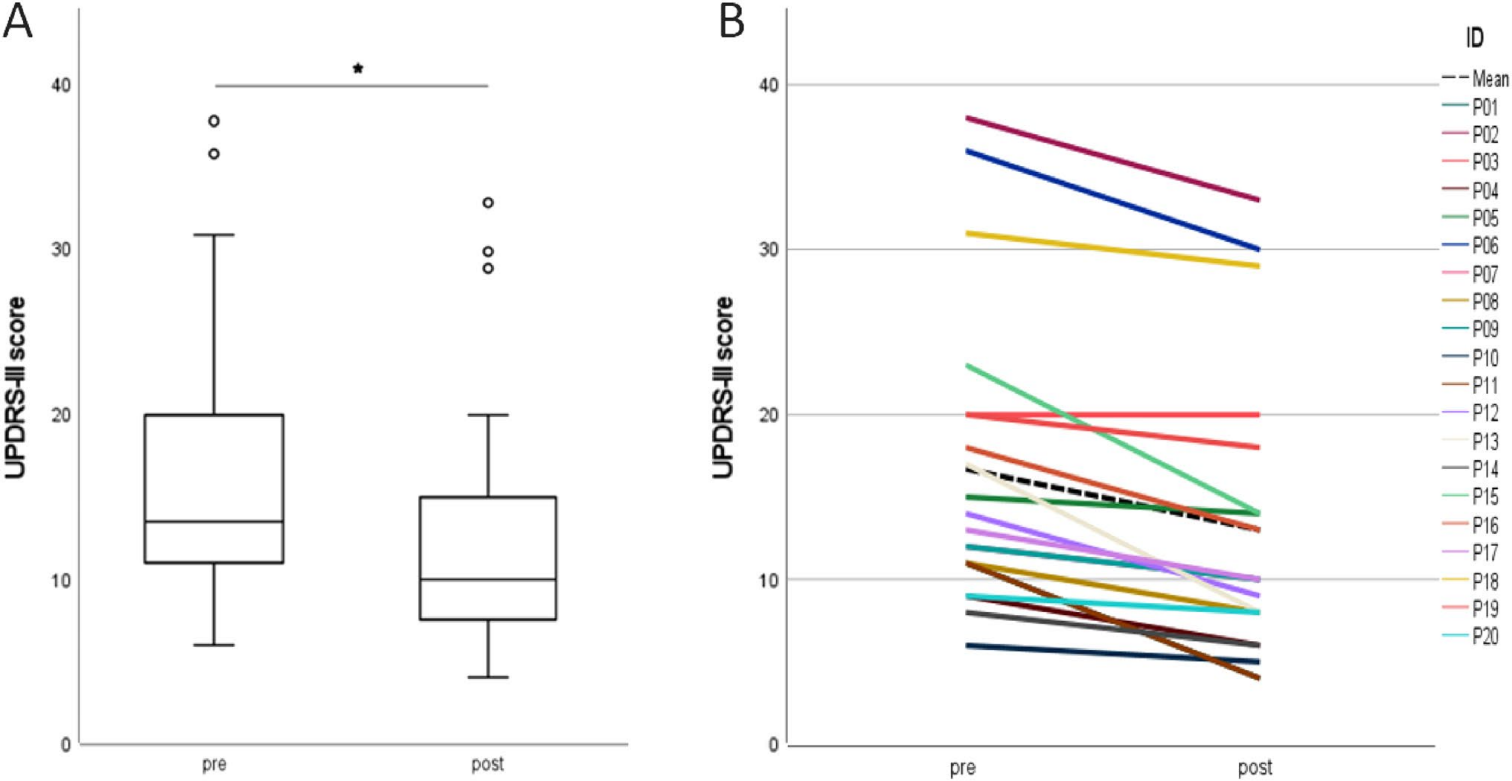
Comparison of Unified Parkinson’s Disease Rating Scale part III (UPDRS-III) total scores pre- and post-transcranial pulse stimulation (TPS).** A** Motor symptoms improved significantly after TPS (*p* < .001; paired *t* test, two sided). Boxplots represent the medians, and the 25th and 75th percentiles, whereas error marks demonstrate the minimum and maximum values. **B** Individual change of UPDRS-III total score after transcranial pulse stimulation (TPS). Each patient is indicated by a different color; the mean value is marked as dashed line. The UPDRS-III total score decreased in 19 out auf 20 patients after TPS

Patient details are summarized in Table 1. UPDRS-III scores (representing the main parameter for patients’ motor symptoms) improved significantly after treatment (pre-TPS: 16.70 ± 8.85, post-TPS: 12.95 ± 8.55; *p* < 0.001; Cohen’s d = 1.38; Fig. 1). Seven patients exhibited an UPDRS-III improvement of at least five points. None of the patients experienced worsening.

**Table 1:** Demographic and clinical characteristics of patients

Pt.no	Disease duration (months)	UPDRS-III	
		**Pre**	**Post**
P01	107	11	4
P02	52	38	33
P03	41	20	20
P04	17	9	6
P05	82	15	14
P06	34	36	30
P07	9	12	10
P08	47	11	8
P09	9	6	5
P10	120	11	4
P11	70	14	9
P12	36	17	8
P13	30	8	6
P14	45	23	14
P15	70	18	13
P16	45	13	10
P17	48	12	10
P18	148	31	29
P19	57	20	18
P20	3	9	8
Mean ± SD	53.5 ± 36.8	16.70 ± 8.85	12.95 ± 8.55

## Discussion

We present a first retrospective investigation of ultrasound neuromodulation as add-on therapy in PD patients on optimized state-of-the-art treatment. It is important to note that the patients represent a heterogeneous consecutive out-patient sample as typical for real-life clinical practice and required for judgement of practical benefits. We find a clear pattern of motor improvement after ten sessions of TPS treatment and lack of clinically relevant side effects, indicating that TPS may be a valuable add-on treatment for motor symptoms in PD. Within the clinical field, a five-point UPDRS change from baseline is considered as the minimal change that represents a clinically meaningful improvement [17]. In our analysis, seven patients were able to achieve or exceed this cut-off score. However, it is important to note that the presented results are not controlled by a sham group. The observed therapeutic success may include placebo effects as previously described in clinical NIBS literature [18–22].

There is evidence that placebo treatment triggers dopamine release in the dorsal striatum, which correlates with placebo-induced improvements in PD symptoms [14, 23]. The anticipation of symptom improvement in response to placebo administration has also been linked to dopamine release in the ventral striatum and activation of the reward circuitry [15, 23, 24]. A landmark meta-analysis of placebo groups in 11 randomized clinical trials in PD [25] revealed a placebo effect of up to 55% with highest placebo response rates in surgical studies involving patients with motor fluctuations. With deep-brain stimulation (DBS), placebo effects reached 39% of active DBS [26].

For more details, we conducted a PubMed search for placebo-controlled NIBS-studies with at least ten PD patients receiving active stimulation. Out of the 16 studies analyzed and covering 445 patients [27–42], 12 found significant improvements in motor symptoms after NIBS intervention [27–38], whereas 4 did not [39–42]. Surprisingly, only 1 study described significant sham effects [35]. This might be due to publication bias or methodological issues [e.g., problems with authentic sham-stimulation (for review, see Braga et al. 2021 [43])] and requires further investigation.

In our study, a certain amount of placebo effect seems likely due to the following reasons: First, placebo responses in non-pharmacological interventions tend to be greater compared to pharmacological studies [44]. Second, placebo responses increase with treatment intensity and duration [45]. Our patients received intensive care over a 2-week period. Third, the likelihood of receiving real treatment versus placebo influences the odds of placebo responses [46]. Fourth, requesting TPS treatment indicates a high expectation level [47]. However, the clear pattern and frequency of motor improvements (19/20 patients improved) render exclusive placebo effects unlikely. From previous TPS investigations which included sham controls and independent neurophysiological data (EEG and fMRI), there is clear evidence for TPS modulation of somatosensory evoked potentials, long-term neuroplastic changes, and long-term improvement of cognitive functions in AD and depression [2, 6, 9, 48]. These findings, together with other clinical data [5], highlight the potential of ultrasound to develop towards a novel add-on neuromodulation therapy.

This is the first demonstration of ameliorating motor symptoms in PD patients using ultrasound stimulation. However, there are limitations to be considered. This was a retrospective analysis of real patient data, and there was no sham control included, and thus, results need to be interpreted with care. Furthermore, the small sample size limits any premature conclusions on the generalizability of the findings. Another crucial consideration in this context is the substantial variability in the efficacy of NIBS demonstrated across clinical PD trials [49]. The considerable heterogeneity of protocols and study populations within the PD-NIBS domain poses a challenge in interpreting the results comprehensively.

## Conclusion

TPS is a promising novel brain stimulation technique. The presented results support and extend the understanding of the safety and efficacy profile of TPS in the treatment of neurodegenerative diseases. Prospective sham-controlled studies with larger sample size are needed to further expand the knowledge on this approach, including long-term effects. However, the findings of this retrospective analysis represent a strong argument to further investigate the value of TPS as a novel add-on therapy for PD.

### Supplementary Information

Below is the link to the electronic supplementary material.

Supplementary file1 (PDF 164 KB)

## Data Availability

The datasets analyzed for the current study are not publicly available due to patient confidentiality and participant privacy restrictions.

## Abbreviations

AD: Alzheimer’s disease
AE: Adverse events
MDS: Movement disorder society
MRI: Magnetic resonance imaging
NIBS: Non-invasive brain stimulation
PD: Parkinson’s disease
ROI: Region of interest
SI: Supplementary information
TPS: Transcranial pulse stimulation
UPDRS: Unified Parkinson’s Disease Rating Scale
VAS: Visual analogue scale
