# Supplementary material for: Novel ultrasound neuromodulation therapy with transcranial pulse stimulation (TPS) in Parkinson’s disease: a first retrospective analysis
Source: J Neurol. 2023 Nov 30;271(3):1462–8. doi: 10.1007/s00415-023-12114-1 (PMC10896933; doi:10.1007/s00415-023-12114-1)
Supplement: Supplementary file 1 — Supplementary file1 (PDF 164 KB) [file 415_2023_12114_MOESM1_ESM.pdf]

Supplementary Information

Table S1: Individual treatment settings and TPS parameter adjustments

ID	Number of TPS sessions	Pulse repetition frequency (Hz)	Deviations from the standard pulse count	Deviations from the standard energy flux density	Days between UPDRS (pre) and first TPS session	Days between last TPS session and UPDRS (post)
P01	10	4	none	none	6	6
P02	10	4	none	2x double stim[1] with 0.2 mJ/mm²	0	0
P03	10	4	none	none	0	0
P04	10	4	none	none	14	61
P05	10	4	none	none	5	25
P06	10	4	none	1x double stim[1] with 0.2 mJ/mm²	21	18
P07	10	4	none	none	49	3
P08	10	4	none	1x double stim[1] with 0.2 mJ/mm²	7	6
P09	10	4	none	none	0	10
P10	10	4	first TPS session with 2000 pulses	1x double stim[1] with 0.2 mJ/mm²	0	0
P11	10	4	first TPS session with 2000 pulses	none	26	12
P12	10	4	first TPS session with 2000 pulses	none	18	6
P13	10	4	first TPS session with 2000 pulses	none	10	4
P14	10	4	first TPS session with 2000 pulses	8 sessions with 0.2 mJ/mm²; 2 sessions with 0.1 mJ/mm²	17	21
P15	10	4	first TPS session with 2000 pulses	2x double stim[1] with 0.2 mJ/mm²	2	0
P16	10	4	none	1x double stim[1] with 0.2 mJ/mm²	14	11
P17	10	4	none	1x double stim[1] with 0.2 mJ/mm²	26	22
P18	10	4	first TPS session with 2000 pulses	1x double stim[1] with 0.2 mJ/mm²	34	18
P19	10	4	first TPS session with 2000 pulses	none	8	9
P20	10	4	first TPS session with 2000 pulses	1x double stim[1] with 0.2 mJ/mm²	24	21

[1] Double stim refers to the administration of two TPS sessions in a single day, as outlined in the Methods.

Table S2: List of patient comorbidities

ID	Diagnosis	Comorbidities
P01	PD	Arterial hypertension
P02	PD	/
P03	PD	Alzheimer's Disease
P04	PD	/
P05	PD	Bilateral presbycusis with hearing aids, Glaucoma
P06	PD	/
P07	PD	Arterial hypertension, Supraventricular extrasystoles, Multinodular Goiter, Chronic bronchitis
P08	PD	Hypercholesterolemia, Mild organic mental disorder
P09	PD	Frontoparietal atrophy, Migraine, Nerve root compression L3/L4, Sudeck's atrophy
P10	PD	/
P11	PD	Mild restless leg syndrome, Mild atherosclerotic macroangiopathy of the carotids, S1 root irritation
P12	PD	Dementia syndrome, Anxiety disorder and moderate depressive episode, Nodular goiter, Epiphyseal cyst
P13	PD	Hypercholesterolemia
P14	PD	Orthostatic dysregulation, Hyperthyroidism, Chronic obstructive pulmonary disease, stage I, Depressive episodes
P15	PD	Mild frontal lobe syndrome, Orthostatic hypertension, Lumbar spinal canal stenosis at L4/L5, Hypacusis, Mild polyneuropathy
P16	PD	/
P17	PD	Supra-aortic sclerosis with thickening of the intima media, Supratentorial subcortical lesions, Mild osteochondrosis C5/C6, C5/C6 Disk bulging, Retrospondylophytes, Constriction of the neuroforamina, Mild constriction of the neuroforamina C6/C7
P18	PD	/
P19	PD	Neurodegeneration with brain iron accumulation
P20	PD	Arterial hypertension
